# Hemodynamic changes under balloon occlusion of hepatic artery: predictor of the short-term therapeutic effect of balloon-occluded transcatheter arterial chemolipiodolization using miriplatin for hepatocellular carcinoma

**DOI:** 10.1186/s40064-016-1880-7

**Published:** 2016-02-24

**Authors:** Yoshiki Asayama, Akihiro Nishie, Kousei Ishigami, Yasuhiro Ushijima, Yukihisa Takayama, Daisuke Okamoto, Nobuhiro Fujita, Koichiro Morita, Hiroshi Honda

**Affiliations:** Department of Clinical Radiology, Graduate School of Medical Sciences, Kyushu University, 3-1-1 Maidashi, Higashi-ku, Fukuoka, 812-8582 Japan; Department of Radiology Informatics and Network, Graduate School of Medical Sciences, Kyushu University, 3-1-1 Maidashi, Higashi-ku, Fukuoka, 812-8582 Japan

## Abstract

To clarify the hemodynamic changes under balloon occlusion of the hepatic artery and to identify predictors of the short-term therapeutic effect (TE) after balloon-occluded transcatheter arterial chemoembolization using miriplatin (B-TACE) for hepatocellular carcinoma (HCC). Tumor variables and angiographic data were collected for 35 nodules (29 patients) with a B-TACE for HCC. Lesions were classified into three groups based on the balloon-occluded CT hepatic arteriography (BO-CTHA) results: Group A, presence of corona enhancement; Group B, absence of corona enhancement; Group C, decreased perfusion or perfusion defect compared to standard CTHA. Objective response was defined as TE3/4, and poor TE as TE1/2, evaluated by response evaluation criteria in cancer of the liver at 1–4 months after the procedure. Univariate analysis revealed that proximal level of balloon occlusion, intratumoral lower CT values immediately after treatment and BO-CTHA findings were significantly correlated with poor TE (p = 0.034, 0.037, and 0.003, respectively). Multivariate logistic analysis identified the Group C as a significant factor associated with the worse short term TE (odds ratio 8.34; 95 % confidence interval 1.49–68.8). Partial or complete perfusion defect on BO-CTHA was an independent factor associated with poor therapeutic effect.

## Background

Transcatheter arterial chemoembolization (TACE) is a widely accepted and effective therapy for unresectable hepatocellular carcinoma (HCC; Llovet et al. 2002). Various anticancer drugs can be used for TACE, including doxorubicin, epirubicin, cisplatin and mitomycin, but the superiority of any particular drug in terms of effectiveness has not been established (Ishikawa et al. 2014). Miriplatin hydrate (Miripla^®^; Dainippon Sumitomo Pharma Co., Osaka, Japan), which has the same diaminocyclohexane structure as oxaliplatin, is a lipophilic derivative that can be suspended in lipiodol (Hanada et al. 2009), became commercially available in 2010. Theoretically, miriplatin is a good agent in terms of its higher solubility, stability in lipiodol, and gradual release within the tumor. However, a 2012 study revealed that the rate of local recurrence in patients treated with miriplatin was significantly higher than that in patients treated with epirubicin with mitomycin (Miyayama et al. 2012). This is due to the high viscosity of miriplatin–lipiodol suspension (Kora et al. 2013). Some researchers have tested creative TACE methods to overcome the high viscosity problem and improve the therapeutic effect (TE); these methods including warming miriplatin (Kora et al. 2013) and balloon-occluded TACE (B-TACE; Ishikawa et al. 2014).

B-TACE was first introduced by Irie et al. (2013). They reported that selective B-TACE with doxorubicin and mitomycin induced dense lipiodol accumulation in HCC nodules. Ishikawa et al. (2014) revealed that B-TACE with miriplatin achieved relatively good local control of HCC (local recurrence rate, 11.1 % at 6 months and 26.2 % at 12 months). The mechanism of this improved local control effect might be explained by the presence of anastomotic vessels, the viscosity of lipiodol emulsion, and the difference in size between the peripheral vessels in normal parenchyma and the vessels feeding into the HCC (Irie et al. 2013). Ishikawa et al. (2014) mentioned that altering hemodynamics might be a main factor under balloon occlusion.

Computed tomography during hepatic arteriography (CTHA) is a sophisticated method for estimating the hepatic arterial flow. We speculated that CTHA under balloon occlusion can provide useful information for the analysis of hemodynamic changes. The aim of the present study was to reveal the relationship between changes of arterial flow in HCC nodules under balloon occlusion and local TE by means of CTHA. We also addressed other predictive factors of local recurrence after B-TACE using miriplatin.

## Methods

### Patients

This retrospective clinical study was approved by the ethics committee of our hospital. The requirement for informed patient consent was waived. From May 2014 to August 2014, 50 consecutive patients with hypervascular HCC who were scheduled to receive B-TACE using miriplatin were evaluated. The exclusion criteria were: (1) death after B-TACE (pneumonia, one case; rupture of duodenal varices, one case); (2) HCCs with a total sum of tumor dias. > 6 cm according to a previous report (Kora et al. 2013; seven cases), additional treatment such as surgical resection, radiofrequency ablation or percutaneous ethanol injection therapy before the follow up CT (seven cases), or chemotherapy without embolization (five cases).

Finally, a total of 29 patients with 35 HCC nodules were enrolled in this study. The clinical characteristics of the study population are summarized in Table 1. All patients underwent pretreatment physical and laboratory examinations, ultrasound, and three-phase dynamic CT or gadoxetic-enhanced MRI. Hypervascular HCC was diagnosed by means of the imaging findings in addition to high serum levels of tumor markers [alpha-fetoprotein (AFP) and d-gamma-carboxyprothrombin (DCP)]. The mean tumor diameter was 16.6 mm (range 9–40 mm).

**Table 1:** Demographic characteristics and pretreatment assessment of 29 patients who underwent B-TACE

Number of nodules, 1/2/3	24/4/1
Age, years	72 (39–83)
Gender, male/female	21/8
Etiology, HBV/HCV/AL/others	5/17/3/4
Child–Pugh class, A/B/C	25/4/0
Albumin, g/dL	3.6 (2.2–4.2)
Total bilirubin, mg/dL	1.0 (0.5–3.29)
Prothrombin activity, %	78 (51–111)
Platelets, ×10^3^/µL	112 (31–219)
AFP, ng/mL	13.7 (2–3791)
DCP, AU/L	39 (9–2043)

Data are median and range in age, albumin, total bilirubin, prothrombin activity, platelet, AFP and DCP

*HBV* hepatitis B virus, *HCV* hepatitis C virus, *AL* alcohol, *AFP* alpha-fetoprotein, *DCP* des-gamma-carboxyprothrombin

### TACE

For each patient, the TACE procedure was as follows. The femoral artery was catheterized under local anesthesia, and a 4-Fr catheter was inserted into the celiac or common hepatic artery. Then a 1.8-Fr coaxial micro-balloon catheter (Logos, Piolax, Yokohama, Japan) was advanced into the hepatic artery that supplied the entire target tumor. CT hepatic arteriography was performed before and after inflation of the balloon. Subsequently, miriplatin–lipiodol suspension was injected followed by gelatin sponge particle (Gelpart; Nippon Kayaku, Tokyo). Miriplatin was prepared by mixing 30 or 60 mg (1 or 2 vials) of miriplatin hydrate in 3 or 6 mL of lipiodol. The maximum dose of miriplatin was limited to 120 mg.

### CTHA

CTHA was performed with an interventional radiology computed tomography (IVR-CT) system (Infinix Celeve/Active, Toshiba Medical Systems, Tokyo). This system is equipped with 16 detector-row CT. The catheter tip was advanced as close to the tumor as possible. Standard CTHA and CTHA with balloon occlusion (BO-CTHA) were performed for the area(s) containing at least one HCC nodule.

The parameters for the scanning were as follows: collimation, 1 mm; reconstruction, 3 mm; pitch, 15: amperage, 300 mA s; kilovoltage, 120 kVp. The CTHA data acquisition began 7–10 s (first phase) and 30 s (second phase) after the initiation of a transcatheter hepatic arterial injection of 10–50 mL of nonionic contrast material (iopamidol, Iopamiron^®^ 150 iodine, 150 mg I/mL; Bayer HealthCare, Osaka, Japan) at a speed of 0.5–2.5 mL/s using the automated power injector. The appropriate injection rate for CTHA was determined to be the maximum injection rate (which basically depended on the vessel caliber) that would not cause a backward flow of contrast material on the hepatic arteriography. The injection rate was the same before and after the inflation of the balloon. The contrast medium was injected until the completion of the scanning of the first phase. Plain CT was performed immediately after the B-TACE. When the plain CT showed the insufficient lipiodol accumulation in the target tumor, we performed the additional TACE without balloon occlusion from another artery in the same session.

### Assessment of the CT findings

**Fig. 1:**
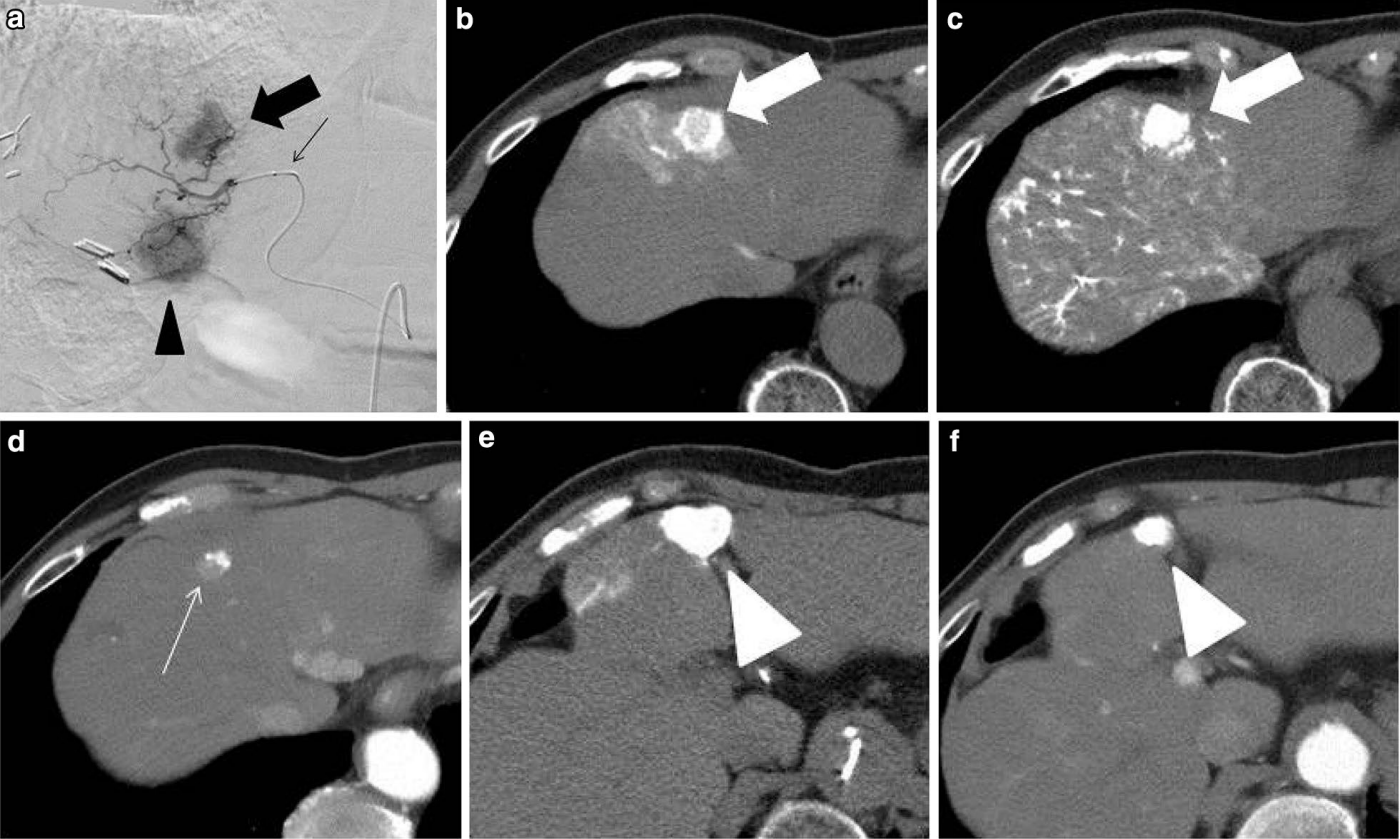
A 73-year-old male with two HCCs (*thick arrow*, *arrowhead*) in segment 4. **a** Digital subtraction angiography (DSA) showed two hypervascular masses (cranial: *thick arrow*, caudal; *arrowhead*). Note the deflated balloon at the tip of catheter (*thin arrow*). **b** Second phase BO-CTHA in segment 4 showed corona enhancement around the tumor. This tumor was classified as Group A. **c** Plain CT obtained immediately after B-TACE showed good accumulation in the tumor, but a CT value measured relatively low at 788 HU, and contrast-enhanced CT at 2 months after B-TACE **d** showed the washout of lipiodol and early enhancement in the tumor (*thin arrow*), indicating poor TE. **e** Second-phase BO-CTHA in segment 4 did not show corona enhancement around the tumor. This tumor was classified as Group B. Plain CT obtained immediately after B-TACE showed good accumulation in the tumor with a high CT value at 1408 HU (not shown). **f** Contrast-enhanced CT at 2 months after B-TACE showed dense lipiodol accumulation in the tumor. The tumor decreased in size without early enhancement

**Fig. 2:**
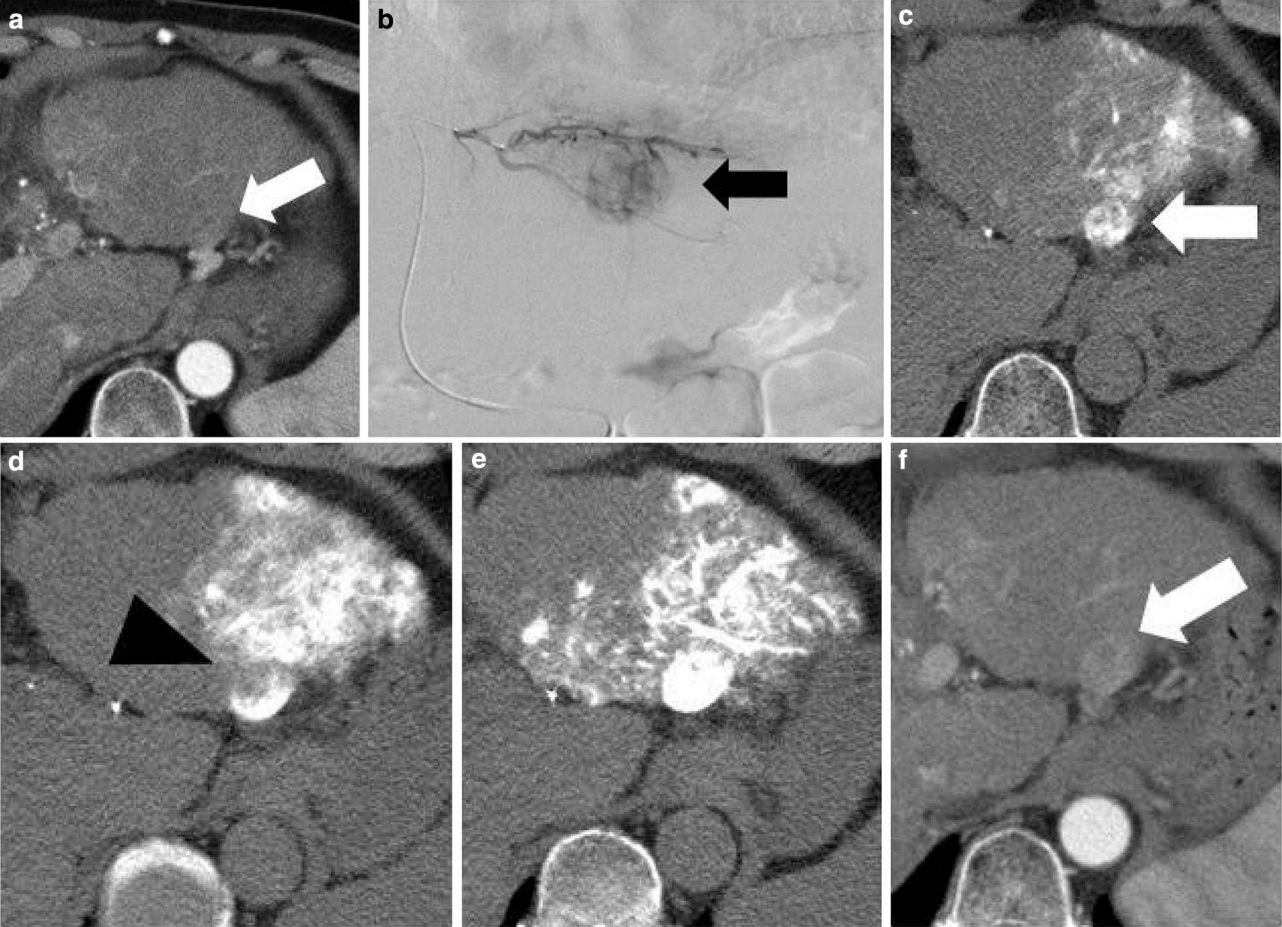
An 67-year-old male with HCC in segment 2. **a** Arterial phase of dynamic CT showed a hypervacular mass (*arrow*). **b** DSA in segment 2 showed a tumor stain (*arrow*). **c** First-phase standard CTHA in segment 2 showed a round hypervascular mass (*arrow*). **d** First-phase BO-CTHA in segment 2 showed a perfusion defect (*arrowhead*) in the tumor. **e** Plain CT obtained immediately after B-TACE showed good accumulation in the tumor with a CT value of 833 HU. **f** Arterial phases of dynamic CT at 3 month after B-TACE showed lipiodol washout and early enhancement, indicating a poor TE. Despite the relatively high CT value immediately after B-TACE, most of the lipiodol was washed out. This is presumably because the inflow from the peribiliary plexus or isolated artery would wash away the lipiodol suspension

Two experienced abdominal and interventional radiologists (AN, YU) blinded to the patients’ information retrospectively reviewed the findings obtained by the standard CTHA and BO-CTHA and the post-treatment plain CT. The tumor locations were classified into two groups: the central portion of the liver (segments 1 and 4) and others (segments 2, 3 and 5–8). All lesions were classified into one of the following groups on the basis of the enhancement pattern observed on CTHA: Group A, nodules that showed high enhancement accompanied by corona enhancement (Ueda et al. 1998) on the BO-CTHA (Fig. 1a–d); Group B, nodules that showed high enhancement without corona enhancement on the BO-CTHA (Fig. 1e, f); Group C, nodules that showed decreased enhancement or perfusion defect on BO-CTHA compared to standard CTHA, regardless of corona enhancement (Fig. 2). Corona enhancement was defined as peritumoral contrast enhancement on the second phase of CTHA (Ueda et al. 1998). The CT value of lipiodol in the HCC nodule was measured by using a region of interest (ROI) in the tumor. The lesion density is presented in Hounsfield units (HUs) on CT.

### Assessment of the therapeutic effect (TE)

The assessment of chemoembolization was evaluated by dynamic CT at 1–4 months after B-TACE. The degree of lipiodol accumulation was classified into four grades according to the response evaluation criteria in cancer of the liver (Kudo et al. 2010). The four TE grades were defined as follows: TE4, 100 % necrosis/size of tumor; TE3, 50–99 %; TE2, up to 50 % or tumor enlargement < 25 %; and TE1, tumor enlargement of 25 % or more, regardless of the extent of necrosis (Kudo et al. 2010). TE is classified into two groups: objective response (good TE), TE3 and TE4; poor TE, TE2 and TE1.

### Statistical analysis

We examined the categorical variables by Fisher exact test. These variables included sex (male, female), Child–Pugh class (A, B, C), AFP (<20, ≥20), DCP (<40, ≥40), previous TACE (yes, no), location of tumor (segment 1/4, others), and level of balloon occlusion (peripheral to the subsegmental branch, segmental to lobar branch). We used the Kruskal–Wallis test to analyze the association between the CTHA findings (Group A, B, C) and the TE; first, we compared the TE among three groups. If a significant difference was obtained, we compared it between each pair of groups using the Chi-square test. Continuous variables such as age and CT value were examined by Student t test. All variables found to be significant in a univariate analysis were entered into the multivariate logistic regression analysis. All statistical analyses were performed with JMP software (version 11, SAS Institute) for Microsoft Windows. Differences at p < 0.05 were considered to be significant.

## Results

The number of TE4, TE3, TE2, and TE1 were 3, 17, 6 and 9, respectively. The results are shown in Table 2. Objective response rate was 42.9 % (15 of the 35 lesions) at 1–4 months after B-TACE. There was no significant difference between the poor (TE1/2) and good (TE3/4) groups in age, sex, Child–Pugh class, AFP level, DCP level, history of previous TACE or tumor location. Thirteen of the 23 tumors who were injected with miriplatin–lipiodol suspension from a peripheral to subsegmental branch showed a good response (TE3/4), whereas 2 of the 12 tumors who were injected with miriplatin–lipiodol suspension from a segmental branch to lobar branch showed a poor response (TE1/2), a significant difference (p = 0.034). The CTHA findings and TE were significantly correlated (p = 0.003). The Group C tumors (Fig. 2) showed significantly poor TE compared to the Group B tumor (Fig. 1e, f) (p = 0.002). Groups A (Fig. 1a–d) and C differed but not significantly (p = 0.075). There was no significant difference between Group A and Group B (p = 0.350). CT obtained immediately after B-TACE showed the lipiodol accumulation in the entire lesions of the all cases regardless of CTHA findings. The CT values of the lesions ranged from 246 to 1407 (mean; 781) and were correlated with the TE (p = 0.037). As shown in Table 3, multivariate logistic regression analysis identified the Group C as a significant factor associated with the worse short term TE bearing an odds ratio of 8.34 (95 % confidence interval 1.49–68.8).

**Table 2:** Therapeutic effect after balloon-occluded transcatheter arterial chemoembolization using miriplatin for hepatocellular carcinoma

	Therapeutic effect		p value
	Poor (TE1/2)	Good (TE3/4)	
Age			
Mean ± SD	73.1 ± 2.1	70.1 ± 2.5	0.366
Sex			
Male	16	11	0.700
Female	4	4	
Child–Pugh class			
A	17	11	0.430
B	3	4	
C	0	0	
AFP (ng/mL)			
<20	12	10	0.928
≥20	8	5	
DCP (mAU/mL)			
<40	8	9	0.315
≥40	12	6	
History of previous TACE			
Yes	13	11	0.721
No	7	4	
Tumor location			
S1 or S4	6	2	0.419
Others	14	13	
Level of balloon occlusion:			
Peripheral/subsegmental	10	13	0.034
Segmental/lobar	10	2	
CTHA finding			
Group A	5	5	0.003
Group B	2	8	
Group C	13	2	
CT value after B-TACE (HU)	711.8 ± 74.2	958.5 ± 85.7	0.037

*AFP* alpha-fetoprotein, *DCP* des-gamma-carboxyprothrombin

**Table 3:** Multivariate logistic analysis for the short term poor therapeutic effect (good vs poor)

Factors	Odds ratio	95 % CI	p value
Level of balloon occlusion (segmental/lobar)	3.59	0.53–33.0	0.191
CTHA finding (Group C)	8.34	1.49–68.8	0.015
CT value after B-TACE (HU)	0.38	0.007–15.0	0.608

*CI* confidence interval, *CTHA* CT hepatic arteriography, *B*-*TACE* balloon-occluded transcatheter arterial chemoembolization, *HU* Hounsfield unit

## Discussion

The therapeutic efficacy of TACE using miriplatin has been controversial. Otsuji et al. (2015) reported that cisplatin and miriplatin had equal efficacy for TACE and transcatheter arterial infusion (TAI) in a randomized controlled trial. Oguro et al. (2012) reported that TACE using a miriplatin–lipiodol suspension yielded worse short term responses than cisplatin–lipiodol suspension. Handa et al. (2014) reported that miriplatin was superior to epirubicin in the short term but inferior in the long term, whereas Miyayama et al. (2012) observed that the local recurrence in patients who were treated with miriplatin was significantly higher than those treated with epirubicin plus mitomycin C, because the high viscosity of miriplatin causes an inadequate distribution in the tumor and early washout.

Ishikawa et al. (2014) mentioned that B-TACE is a promising method for improving treatment with miriplatin. B-TACE, originally developed by Irie et al., achieved good accumulation of lipiodol emulsion in the HCC nodules. They reported that in the setting of a balloon occlusion, lipiodol emulsion ceased to flow in the peripheral vessels supplying liver parenchyma but still continued to flow into HCC nodules in the majority of the cases (Irie et al. 2013). In some cases, lipiodol emulsion did not show stagnation in the peripheral vessels supplying the liver parenchyma, leading to poor local control (Irie et al. 2013). Ishikawa et al. (2014) speculated that the mechanism underlying the good accumulation of lipiodol is essentially an alteration of the hemodynamics under balloon occlusion.

**Fig. 3:**
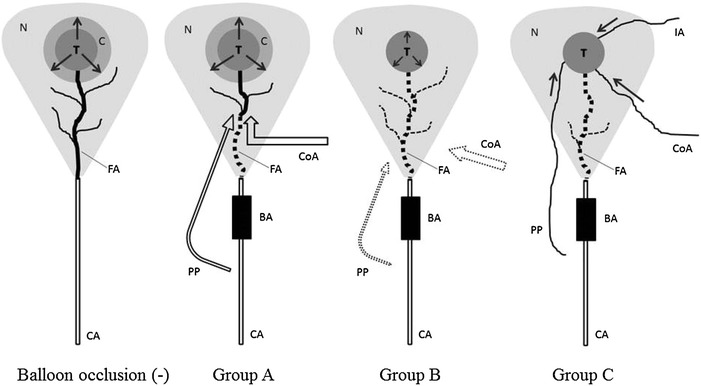
Changes in the intrahepatic arterial flow with and without balloon occlusion. Without balloon occlusion, the feeding artery flows into the tumor and drains out to the hepatic parenchyma, forming corona enhancement. In Group A, the feeding artery is reconstructed by the peribiliary plexus and communicating arcade distal to the balloon occlusion. The arterial inflow was kept in the tumor, and the intra-arterial pressure around the tumor was preserved. Corona enhancement was thus observed. In Group B, the collateral arteries such as the peribiliary plexus and communication arcade do not develop enough to maintain the intratumoral arterial pressure. Corona enhancement is not present in this group. In Group C, collateral arteries including the isolated artery, peribiliary artery and communicating arcade feed the tumor entirely or partially. These flows do not contain contrast material, and thus CTHA shows decreased perfusion or perfusion defect. *CA* catheter, *FA* feeding artery, *N* the area of contrast distribution in noncancerous liver parenchyma, *T* tumor, *C* coronal enhancement, *BA* balloon (inflated), *PP* peribiliary plexus, *CoA* communicating arcade, *IA* isolated artery

Three types of hepatic artery play an important role in the hemodynamic change under balloon occlusion (Irie et al. 2013): peribiliary plexus (Cho and Lunderquist 1983), interlobar communicating arcade (Tohma et al. 2005), and isolated artery (Ekataksin 2000) (Fig. 3). In Group A, the corona phenomenon (Ueda et al. 1998) was observed in the second phase of BO-CTHA. We think that in this group, the arterial blood pressure did not decrease due to prominent collateral arterial flow such as that in the peribiliary plexus and interlobar communicating arcade beyond the occluded portion or due to inadequate balloon inflation. Thus, the intratumoral arterial blood pressure overcomes the portal venous pressure surrounding the tumor, resulting in the corona phenomenon.

The corona phenomenon was not observed in Group B, in which the intratumoral arterial pressure probably decreased and might be nearly equal to the portal venous pressure in the surrounding tumor. In such a condition, the corona phenomenon cannot be seen. In the present study, even though no significant difference was observed in local control between Groups A and B, there was a tendency for Group B to show better local control than Group A. Lipiodol suspension could be pushed into the tumor and into the area of corona enhancement more easily in Group B.

In Group C, we suspect that collateral tumor vessels such as the isolated artery feed directly the tumor instead of the original feeding artery under the condition of balloon occlusion. In the beginning of the infusion, most of the lipiodol suspension runs through the artery in the noncancerous liver parenchyma but instantly ceases to flow, and then it flows into the original feeding artery, leading to a good accumulation of lipiodol suspension in the tumor immediately after treatment. However, because collateral tumor vessels may develop well in such tumors, the lipiodol would wash out rapidly.

In the present study, the TE was worse than that in a previous evaluation (Ishikawa et al. 2014) showing an only 11 % overall local recurrence rate at 6 months. This might be due to the development of the collateral arterial flow during the BO-CTHA. Taking time to perform BO-CTHA could allow the development of the collateral arterial flow into the tumor even for just a short while, causing early washout of lipiodol after treatment.

There were no significant differences between the good TE group and the poor TE group in age, sex, Child–Pugh class, previous TACE, or tumor location. As for tumor location, even though no significant difference was observed, 6 of 8 tumors (75 %) in the central portion (segments 4 and 1) showed a poor therapeutic effect. This is probably because the catheter access for the feeding artery is difficult in segments 4 and 1 in general, and migration or spillover of the drug into the communicating arcade is frequent.

With regard to the occlusion level of balloon, our proximal occlusion cases (segmental or lobar artery) showed poor TE. This may be because a large amount of drug was delivered in the noncancerous liver parenchyma, thus the amount of drug into the tumor would be smaller compared to the cases treated with the infusion of drug from a peripheral to subsegmental artery and because the intratumoral arterial pressure would not decrease enough due to collateral flow. Finally, the plain CT value immediately after treatment was a predictive factor of TE in our study, which is in concordance with the previous report (Ishikawa et al. 2014).

The present study has several limitations. First, the CTHA was not performed for all of the lesions for each case. In the cases of multiple lesions, the largest lesion was selected for CTHA, in principle. This could have caused lesion selection bias. Second, the patients who had undergone chemolipiodolization without embolization using gelatin sponge particles were excluded from this study, and this could also have caused patient selection bias. Third, the viscosity is different between the contrast material and the lipiodol suspension. The distribution pattern of contrast material would not correspond to that of the lipiodol suspension precisely. Furthermore, CTHA is a complicated method and involves large radiation dose. However, we believe that it would be of benefit to understand the hemodynamic changes in the tumor under the balloon occlusion of the hepatic artery. Fourth, we did not evaluate overall survival or long term TE. We consider, however, that the short term TE may be directly related to prognosis of the patients. Fifth, the number of patients was relatively small.

In conclusion, the intratumoral arterial flow can change, presumably due to a collateral pathway under balloon occlusion. B-TACE for HCC lesions showing decreased perfusion or perfusion defect on BO-CTHA compared to standard CTHA can be expected to achieve only a poor short-term therapeutic effect as well as a proximal level of balloon occlusion and lower CT value immediately after treatment. In the multivariate logistic analysis, decreased perfusion or perfusion defect on BO-CTHA was found to be an independent factor associated with poor therapeutic effect.
